# What Happened to the Foundations of Eurasian Health Governance? Research Initiatives for Health Security Capacity Building

**DOI:** 10.1007/978-3-030-42775-7_9

**Published:** 2020-02-13

**Authors:** Ole Döring

**Affiliations:** OSCE Academy, Bishkek, Kyrgyzstan; Berlin, China

## Abstract

When China launched the Belt and Road Initiative (BRI), or New Silk Road Initiative, in 2013, its mind was set on the first stage of globalization with so-called ‘Chinese characteristics.’ These characteristics, also known in general as China’s ‘third way,’ indicate China’s approach, which is to learn innovation rather than copy or follow tradition, in order to hardwire businesses connections to Southeast Asia, Europe and Africa. After six years of breathtaking construction of infrastructure for connectivity, roads and bridges, market interfaces and business interlaces, the overall activities are readjusted, from groundwork toward sustainable activities that propagate social goods and emphasize the soft skills for human flourishing, and generate the translational capacities for cultural engagement.

## Background

When China launched the Belt and Road Initiative (BRI), or New Silk Road Initiative, in 2013, its mind was set on the first stage of globalization with so-called ‘Chinese characteristics.’ These characteristics, also known in general as China’s ‘third way,’ indicate China’s approach, which is to learn innovation rather than copy or follow tradition, in order to hardwire businesses connections to Southeast Asia, Europe and Africa. After six years of breathtaking construction of infrastructure for connectivity, roads and bridges, market interfaces and business interlaces, the overall activities are readjusted, from groundwork toward sustainable activities that propagate social goods and emphasize the soft skills for human flourishing, and generate the translational capacities for cultural engagement.^1^ Ultimately, sustainable prosperity and peace become the desired gems. The craft that is now assigned to provide a sense of orientation and the means for deliberate planning is science.^2^

In the light of the 2020 dramatic Covid-19 Pandemia, this timing, however awkward, indicates an opportunity to consider germs. A look at geography from the viewpoint of microbes displays Eurasiafrica as a massive opportunity for all kinds of microbes and bacteria to prosper without control. The enormous salt-water barriers that block pathways to other continents are rendered inconsequential. Opportunities to adapt and procreate, owing to removed barriers and increased traffic between East and West, North and South, continue to excel. An ever-increasing air-mobility is destined to pave the way for the next wave. Regional stability, including security matters, economic development and the foundations of wealth are at stake. The OSCE region is prominently affected by this flow. This research endeavor elaborates on how policy makers, physicians, scientist, scholars and activists are prepared to prevent or manage the next epidemic or pandemic outbreaks across this vast region.

In 2011, Paul Unschuld and this author initiated preparations for a consortium that would eventually generate a roadmap, theoretical foundations and the methodological framework to describe a governance system that could address these issues, within a Eurasian context: the Eurasia Health and Disease Survey (EAHDS). More than just a quantitative survey in public health, it would provide a tool kit for preventive measures, embedded monitoring and pathogen detection technology, early intervention points and governance interfaces. It would inform protocols and technical, as well as social, legal and ethical, standards applying to security in general. Thereby, it anticipated not only the BRI but also the Global Health Initiative of 2013, and in that, it generates a multidisciplinary, transnational approach considering and cross-linking the social, environmental, political, commercial and other determinants of health in a ‘global’ discourse.^3^ Admittedly, none of the foreseeable health and security issues could be immediately mitigated with this framework. However, without such provisions, it will become much more difficult for governments and non-governmental agencies to conceive, align and orchestrate the due measures to act ‘in the unlikely event’ of a major catastrophe.

## Rationale

Under the Charité Medical University’s Institute for the History, Theory and Ethics of Chinese Life Sciences, and directed by Paul Unschuld in Berlin, a public health project center was established to function as a facilitating base for EAHDS. The project outline explained how the Eurasian continent is not only a geographical but also a physiological unit. The land bridge from the Atlantic to the Pacific covers the whole OSCE region. It is gradually reshaping the old frontiers for trade and transport, thereby opening up new perspectives that also have significant consequences for the health and security sectors. In this view, it will be possible to systematically address international and cross-border issues of diplomacy, technology transfer, epidemiology and public health in a scientific way.

With the growing interdependence between formerly disconnected regions, such as Western Europe, Russia, Kazakhstan and China, the exchange of people, goods and knowledge—and also the accompanying disease germs—is increasing as seen during the Covid-19 Pandemia in 2020. In parallel, opportunities present themselves: to explore epidemiological interfaces and transactions, and the opportunity to address the urgent need for transnational cooperation on understanding, communication, prevention and crisis intervention and monitoring.

Researchers, activists, policy makers and the health and security sectors across these countries and across disciplines are encouraged to support research and take action with transnational and regional problem-solving mechanisms to cover and address all these issues through one key rationale. Due to the historical conditions of the twentieth century, namely the Cold War and the Iron Curtain blocking this route, the necessary structures and also the required knowledge to benefit from the new situation are amiss. Against this background, the EAHDS project appreciates the potential of existing bilateral cooperation, e.g., European and Russian, or Russian and Chinese, or Eurasian and Chinese governments and scientists in the field of health and security. However, the existing structures and forms of cooperation should have and can now be transformed into a system that should cover the entire space, extending from Berlin to Novosibirsk and Astana to Beijing. This would, of course, include other centers such as Vienna and Warsaw, and even Addis Ababa or Accra in the future. For reasons of feasibility, however, the initial focus of EAHDS was set on the Berlin–Moscow–Astana–Beijing axis, to explore the possibilities of such a project.

## Health Security Across the OSCE Region

Health security offers a neutral, and at the same time, promising framework for closer cooperation, because it aligns the interests of different states and offers true win-win potential. Health care is politically, economically and technologically relevant and foundational. The first phase therefore would explore questions of determining the basic health conditions, such as disease spread pattern mapping, institutional infrastructures outlines and state-of-the-art description in order to define desirables and priorities. As for a pilot study, it seemed reasonable to confine ourselves initially to focus a few problem areas which afflict, if not endanger, the Eurasian continent as a whole, in terms of pathogenic potency. Tuberculosis, HIV-AIDS and influenza were named as three closely linked areas of health, all prevalent across the entire land mass and, most importantly, transported in all directions. A scenario of an outbreak of a mutation of the SARS and Corona virus, with the velocity of Ebola, would be working in all directions and could serve as a hypothetical horizon to describe potentially realistic future events. In the first step, regional incidences and the prevalence of these three diseases would be documented as known, the affected populations described, official structures and authorities in charge in the participating states for monitoring mapped and the corresponding challenges addressed. These challenges would include social and cultural/ethical similarities or differences in dealing with these health problems among all relevant administrations. In these different constituencies, the transit and end-point countries, the relevant cultural key concepts such as ‘health,’ ‘risk,’ ‘self-responsibility,’ ‘solidarity,’ 'cooperation,’ ‘aid,’ ‘destiny,’ ‘individual’ and ‘science’ may have different meanings and can be formulated in ways that require not just translation, but interpretation and even negotiation over practical implication and priorities.

The differences can also impact the structures of the respective health systems, since different values are associated with different norms. The related standards cannot simply be prescribed and implemented top-down, as their success depends on compliance and sometimes adherence, which only works out when actors ‘see the point’ and share it within the horizons of their own experience. In concrete terms, the respective social structures and economic trends of the health governance systems in the participating countries must be accounted for. It includes but goes beyond the question of existing emergency crisis plans and the regional means of data collection, the existence of databases in the health sector and other relevant data related to security issues. The local scientific knowledge about and identification of possible biological agents or other pathogens that can spread along Eurasian transport routes, together with social skills in communication and collaboration, are crucial. A methodical question is, what types of pathogens will particularly benefit from increased flows of goods and traffic? What is the variability of pathogens with different pathogenic degrees of individual strains in the Eurasian space? How will they spread under epidemic conditions? Does the analysis of previous pandemics in the regions provide clues that are valuable for the future, for governance and for risk prediction?

Difficulties that arise in the event of an emergency or a crisis, beyond technical or administrative cross-border interaction, can be estimated, for example, from the different retrospectives of the SARS epidemic by European and Chinese representatives, who can now invite Kazakh experts to learn and contribute. In 2002/2003, Chinese statements suggested a belief that SARS only caused low death rates where traditional Chinese medicine had been applied, but much higher mortality where modern medicine was used. This, obviously, contradicts the assessment not only of Western scientists, but also of Chinese scientists, such as researchers at the Beijing Genomic Institute (BGI) who were the first to decipher whole genome sequence of four SARS viruses in early 2003,^4^ but had to hold back making it public for political reasons. As far as China is concerned, relevant lessons seem to have been learned, according to the policies in dealing with the fatal pneumonia-variant caused by a corona virus originating in Wuhan that started to spread in the winter of 2019/20. Reminiscent of the SARS crisis in 2002/03, this variant was found to be transmitted across species into human systems. This potentially posed another serious challenge to Eurasian governance infrastructures, and China’s President Xi Jinping decreed publicly that the virus must be ‘resolutely contained’ and transparently observed in order to secure the trust and collaboration of the people and that all cadres must make ‘the safety of people’s lives and their physical health’ the top priority.^5^ However, between Wuhan and Paris, different interpretations in health-related anthropologies, belief systems and social practices can have severe health and security impact but cannot be discussed only during or after an emergency. The cultural priorities and social standards must be resolved in advance. On this basis, the complex issues of how to harmonize legal, administrative or technical protocols and norms in order to make them work or even be mutually supportive across Eurasia can be addressed. Again, this raises questions about cross-border interaction, when cooperation depends on mutual trust and understanding.

This research would be initially described as a basis for a structural inventory. Then, the most important action areas for transnational public health and security development would be mapped. Sustainable long-term repositories for knowledge, problem-solving and cooperation competence and advice for business, health and research policy could be informed and built up. The aim of the sub-project sketched here was to prepare an IT-supported crisis management system that is efficient when it is needed.

1. It is already clear from these brief remarks that only a large, interdisciplinary approach can generate insights that respond to the complex themes at hand and make this project a success. Disciplines and faculties to be involved to some extent include public health, epidemiology, demography, geography, cultural studies/medical anthropology, medical sociology (risk and communication research), political science (and analytical methods such as genomics and bio-informatics), governance and translational sciences, all of which are perhaps the central areas of expertise. In order to work toward a coherent map and methodology, meta-studies and reflections from Global Health, theory of science and philosophy (ethics and language) would be needed. Moreover, experience from international non-governmental bodies and agencies such as WHO, Red Cross/Crescent and GIZ should be incorporated, as well as innovative science-technology developers, especially in the area of early detection and logistics.
2. Both parts, the groundwork study and the case scenario, would be combined with a theory and technology development modeling project. The key question to be examined for such a scientific research program was then formulated in a hypothesis: Can central functions of swarm intelligence be translated and technically reproduced for cross-border governance tasks? The swarm, as a model, offers several fundamental characteristics of rational collaborating social entities (fish and birds) that does not depend on cultural or social specialization but leaves the option to employ such soft power factors as supplementary resources for added benefit. What technical, infrastructural, communicative and, if applicable, cultural conditions must be met in order to discuss such a project not only academically but with the concrete aim of providing recommendations for infrastructure development and regulatory collaboration across Eurasia? Obviously, IT- and AI-supported new technologies would be designed according to the purpose of such an infrastructure, with bio-sensitive monitoring propensities, semi-autonomous programming and interactive properties.

The described research agenda, especially for the modeling of governance interfaces and IT infrastructures, was seen as an unprecedented and genuinely innovative initiative. It would support the objectives of the EAHDS in a comprehensive and holistic manner, combining coherence of governance measures with aligned trans-disciplinary work toward an integrated module-based Eurasian infrastructure technology. This project acquired a conceptually driven, empirically informed design including elements of public health, governance, information technology, artificial intelligence (bio-robotics) and translational philosophy. It was prepared to build on structurally helpful approaches such as international health regulations and monitoring agencies, to further develop existing governance infrastructures at national and transnational levels and to rebuild a middle level along the sensory-causal interfaces along the Eurasian landmass.

## Measures and Indicators

The Institute for the History, Theory and Ethics of Chinese Life Sciences at the Charité took over the coordination of the program agenda. In particular, over several years it cooperated with institutes in the Fraunhofer-Gesellschaft, as well as with the Institute for Informatics, Biometry and Epidemiology at Ludwig-Maximilians-University Munich and others.

1. Among the measures to roll out the program step by step was a project application, ‘Partnerships for sustainable problem solving in emerging and developing countries—Research for development,’ a pilot action for partnerships in science, research and education with the countries of Central Asia and the south Caucasus, responding to a call from the German Federal Ministry of Science and Technology, submitted in 2012. The proposal was signed by members of the consortium, including Fraunhofer Institute for Systems and Innovation Research ISI, Fraunhofer Institute for Interfacial and Bioprocess Engineering IGB, Molecular Biotechnology MBT, Fraunhofer Institute for Applied Information Technology FIT, Kazakh Medical National University, Centers for Disease Control and Prevention (EU CDC), Society for International Cooperation (GIZ) and the EU Project Infectious Disease Prevention and Control.

The first international partner country at the focus of this feasibility study was Kazakhstan. Located at the core of Eurasia and part of notable modernization policies, Kazakhstan was chosen as a country of origin, transit and end point, where significantly different cultural conditions apply, as compared to Germany. Such differences also feed into security dimensions and affect the structures of the respective health systems. Especially, Kazakhstan differs in terms of the concrete practice in dealing with epidemics, since different values are associated with different norms and as a result with their own order. Kazakhstan had one of the highest prevalence rates of tuberculosis in the WHO region of Europe. At the same time, it was a transit region for pathogens in Central Eurasia, a region where the mobility of people and goods is rapidly increasing. Reported cases of tuberculosis in Kazakhstan have been declining since the early 2000s, but the number of cases of multi-drug-resistant tuberculosis was increasing, as in other Asian countries, such as China.

In this context, the consideration encompassed the respective general strategic interests, incentives, economic trends and special developments in the health systems, the question of the existence of emergency crisis plans and the regional conditions for data collection, or the existence of databases, in the health sector, as well as communicative and cooperative peculiarities. Knowledge about possible biological agents or other pathogens that can spread along the Eurasian transport routes via Kazakhstan is crucial. Other epidemiological problem areas are co-infections with HIV and diseases in hard-to-reach populations, e.g., migrants. Governance-related collaboration seemed feasible and promising because an infrastructure for efficient disease control was under construction, as Kazakhstan had established a Center for Disease Control.

(2) At the interface of health governance and bio-security, with a special interest in cross-border collaboration and relief measures, as part of a regional strategy for prevention, control and early intervention, the availability of well-equipped human resources, together with academic and scientific institution building, inspired an initiative to frame and support measures, such as the above-mentioned research, with a 2016 proposal to establish a multi-center New Silk Road University (NSRU). Considering that particular attention should be paid to early prevention, disaster relief and disaster medicine, in the context of BRI, cooperation would benefit from a network of standardized education hubs, in terms of stability, quality, sustainability and outreach.

In 2016, a building bearing the programmatic name of Robert Koch, at Charite Universitätsmedizin Berlin, was available as a central unit at that time. Other candidate satellite and sister centers were identified or ready to collaborate in the region. The first strategic aim of this NSRU core was to train an international management based on the sciences, which are directly related to the realities of the growing exchange of goods across Eurasia and thus to the objectives of the BRI project. This properly ambitious institution was conceived as a space to think ahead, in order to become a strong and independent partner for experts and institutions to gather strength and momentum while China continues to make the BRI real.

A university network suggests itself as an academic and logistic base for an IT-supported crisis management system that is efficient and functional when emergency calls and continuously learning when it does not. The pilot projects described here, the EAHDS and NSRU, point to a constructive and responsible direction for the future. Meanwhile, any other interested foundations, academic institutions state authorities and companies with a clear humanitarian vision and ambitions for holistic quality are called upon to support this project and to work with the scientific project initiators to find ways to open the Eurasia health policy area.

It is true that, ‘science and epidemic control should not be linked with politics,’ as Gabriel Leung of Hong Kong’s WHO Collaborating Centre for Infectious Disease Epidemiology and Control was quoted saying with reference to the Wuhan outbreak.^6^ However, politics should be obliged to support the ability and freedom of science to serve for Global Health by all means necessary. This applies to the entire region, disregarding national borders, and to the joint resources from all trans-disciplinary fields of knowledge. The SARS-related strain, the Covid-19 from a wet market in Wuhan, fortunately, does not seem to bear the infectiousness of Ebola that could change any time and generate a cascade of security risks which could be prevented with some foresight and investment.

## Footnotes

1 Döring (2018).

2 Masood (2019).

3 Bonk et al. (2018).

4 Bi et al. (2016).

5 Zheng (2020).

6 Zheng (2020).
